# Development and first results of a dedicated chronic total occlusion programme

**DOI:** 10.1007/s12471-019-01348-2

**Published:** 2019-12-03

**Authors:** H. W. van der Werf, P. J. Vlaar, P. van der Harst, E. Lipšic

**Affiliations:** 1 Department of Cardiology, University Medical Centre Groningen, University of Groningen, Groningen, The Netherlands; 2 Department of Cardiology, Catharina Hospital, Eindhoven, The Netherlands

**Keywords:** Chronic total occlusion, J‑CTO score, Percutaneous coronary intervention, Recanalisation, Target vessel revascularisation

## Abstract

**Objective:**

To describe the development and first results of a dedicated chronic total occlusion (CTO) programme in a tertiary medical centre.

**Background:**

Because of the complexity and the increased risk of complications during percutaneous coronary intervention (PCI) for CTO, it is essential that less experienced and evolving CTO centres perform regular quality analyses.

**Methods:**

We therefore performed analyses to describe the results during the first 3 years of a dedicated CTO programme at a high-volume PCI centre. In addition, we discuss the strategies employed to develop such a programme.

**Results:**

A total of 179 consecutive patients undergoing 187 CTO procedures were included in the study. The complexity of the CTO lesions increased from a mean J‑CTO (Japanese Multicentre CTO Registry) score of 1.3 in 2015 to 2.1 in 2017. In the majority of cases, the antegrade wire escalation technique was performed. Final technical success rate was 78.5% in 175 patients with a single CTO and 80.2% of all 187 CTO procedures. No peri-procedural or in-hospital deaths occurred. One peri-procedural myocardial infarction occurred. Cardiac tamponade occurred in 2 cases, both managed by pericardiocentesis. No urgent cardiac surgery was necessary. Survival and revascularisation rates at 30 days and 1 year were excellent.

**Conclusion:**

Following initiation of a dedicated CTO programme, using up-to-date techniques and strategies, procedural and clinical outcome were comparable with current standards in established centres.

## What’s new?

- The introduction of drug-eluting stents, improved techniques (including the hybrid approach) and dedicated chronic total occlusion (CTO) devices have led to a marked increase in procedural success rates of CTO procedures.
- Because of the complexity of CTO procedures, dedicated CTO teams are necessary to achieve adequate success rates with acceptable complication rates.
- During the first years of our dedicated CTO programme the technical and procedural success rates were in accordance with current standards and tended to increase following implementation of the hybrid algorithm.

## Background

Chronic total occlusions (CTOs) are present in around 15% of patients with significant coronary artery disease and are associated with an adverse clinical outcome [1]. Because of low technical success rates and an increased risk of procedural complications, patients with CTOs have historically been referred for surgery or offered medical treatment only. The introduction of drug-eluting stents, improved techniques and dedicated CTO devices have led to a marked increase in procedural success for percutaneous coronary intervention (PCI) for CTOs [2].

Observational studies have reported that successful recanalisation of CTOs can improve angina and quality of life as well as left ventricular ejection fraction (LVEF) and survival [3]. However, randomised controlled trials examining the outcome of CTO-PCI are limited and not definitive [4–7].

Current international guidelines state that the treatment of CTOs may be considered analogous to the treatment of non-CTO lesions [8, 9], which means that a CTO-PCI is indicated in patients with persistent symptoms despite optimal medical therapy (OMT) or in those with a large ischaemic CTO territory. In cases of reduced LVEF with regional wall motion abnormalities in the CTO territory, objective evidence of viability should be present.

Because of the complexity of the procedure and the increased risk of complications during CTO-PCI, it is essential that less experienced and evolving CTO centres perform regular quality analyses. We therefore performed an analysis to describe the first results of a dedicated CTO programme in a tertiary medical centre. In addition, we discuss the strategies employed to develop such a programme.

## Methods

### Development of a CTO programme

Our dedicated CTO team was initiated in 2014 by two interventional cardiologists (H.W. van der Werf and E. Lipšic), who have both performed more than 1000 PCI procedures in the last 5 years. The operators and participating catheterisation laboratory staff were trained by means of educational programmes and on-site proctorship of physicians (by established experts in CTO revascularisation). The CTO operators organised weekly meetings to determine the appropriateness of indications for CTO-PCI, necessary additional examinations and a procedural strategy for each patient. Every week 1 day was reserved for scheduling CTO cases by a 2-operator/case policy. Procedural guidelines were also established related to antithrombotic therapy, iodinated contrast exposure and ionising radiation exposure.

All patients in whom a CTO-PCI was attempted were registered for quality issues. The research complied with the Dutch law on Medical Research in Humans and local research guidelines.

### Patients

The study population consisted of patients who underwent a PCI at the University Medical Centre Groningen in the period from January 2015 to December 2017.

All patients with a CTO treated by physicians of the newly developed CTO team were included in this study. Patients undergoing unplanned CTO procedures by other physicians were not included.

### Procedural details

The indications for performing a CTO-PCI were persistent symptoms under OMT and reversible myocardial ischaemia/viability on non-invasive imaging.

We used bilateral arterial access routinely with a preference for (one) radial artery. At the start of the programme in 2015 mainly antegrade procedures were carried out. From January 2016, the hybrid algorithm (including retrograde wiring through collaterals or grafts and use of device-assisted dissection and re-entry) became common practice.

Local protocols and standard contrast reduction methods were used for the prevention of contrast-induced nephropathy. As radiation exposure is higher during CTO-PCI, the radiation dose was minimised by several additional methods. Extended radiation shields and drapes were used during procedures. Further, a low-dose fluoroscopy protocol was used and the frame rate per second was lowered to 7.5 instead of 10.

Adjunctive therapy included intravenous heparin during the procedure to achieve an activated clotting time (ACT) of 300–350 s during antegrade procedures and >350 s during retrograde procedures. ACT measurement was performed every 30 min. Acetylsalicylic acid was administered prior to the procedure and continued indefinitely. Clopidogrel was given with a loading dose of 600 mg before the start of the procedure and was continued at a dose of 75 mg for at least 12 months after stent implantation. Following uncomplicated PCI, patients were discharged home the same or the following day.

### Data collection

At the start of the CTO programme all patients in whom a CTO-PCI was attempted were prospectively registered for quality issues. Baseline, angiographic, procedural and outcome data were collected and managed using REDCap (Research Electronic Data Capture) electronic data capture tools hosted at the University Medical Centre Groningen [10].

### Definitions

CTOs were defined as a lesion of a native coronary artery that exhibited Thrombolysis in Myocardial Infarction (TIMI) antegrade flow grade equal to 0 for at least 3 months. The J‑CTO (Japanese Multicentre CTO Registry) score was calculated as previously described [11]. Occlusion length was estimated from the angiographic image using a bilateral arterial approach or the stent and balloon length as reference. Presence of calcification in the CTO segment was determined by fluoroscopy without contrast injection.

Antegrade wire escalation (AWE) was defined as antegrade wiring from true-to-true lumen with increasing guidewire tip loads and penetration force if needed. Subintimal tracking and re-entry and limited antegrade subintimal tracking techniques were defined as wire- and device-based antegrade dissection re-entry techniques. Any retrograde approach to the distal CTO cap is considered a retrograde procedure (including retrograde wire escalation (RWE) and dissection re-entry).

Peri-procedural complications included life-threatening and major bleeding (Bleeding Academic Research Consortium criteria), major cardiac complications (including sustained ventricular arrhythmia, coronary dissection, coronary perforation, septal haematoma, cardiac tamponade as well as intracoronary thrombus formation or air embolism) and access site complications.

Myocardial infarction, both in hospital and at 30-day follow-up, was considered to have occurred if at least two of the three following criteria were met: (1) prolonged chest pain ≥20 min, (2) enzyme changes (more than double the upper normal limits of creatine kinase (CK), CK-MB, or relative index), (3) ST-T-wave changes or new Q‑waves on serial electrocardiograms indicative of myocardial damage. Target vessel revascularisation (TVR) was defined as any repeat percutaneous intervention or surgical bypass of any segment of the target vessel. Planned second attempts at revascularisation of CTO lesions were not scored as TVR.

The primary endpoints of this study were technical and procedural success. Technical success was defined as successful CTO revascularisation with achievement of <30% residual diameter stenosis within the stented segment and restoration of TIMI flow grade 3 antegrade. Procedural success was defined as technical success with no procedural major cardiac adverse events. The secondary endpoints included the occurrence of peri-procedural and in-hospital complications, MI, TVR, and mortality at 30-day and 1‑year follow-up.

### Statistical methods

Statistical analyses were performed using SPSS version 23 (Armonk, NY, USA: IBM Corp.) for Windows. A two-tailed probability value of <0.05 was considered statistically significant. Descriptive data are presented as numbers with percentage, as mean with standard deviation or as median with range. Comparisons across the first years of the CTO team were performed by the Pearson chi-square test for categorical variables, one-way ANOVA for normally distributed continuous variables and Kruskal-Wallis test for non-normally distributed continuous variables.

## Results

### Baseline characteristics

**Fig. 1:**
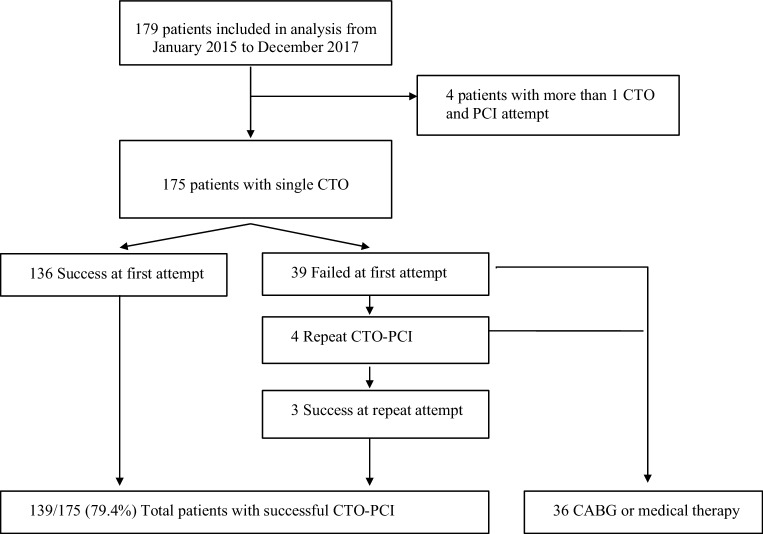
Flow diagram of inclusion criteria. *CABG* coronary artery bypass graft, *CTO* chronic total occlusion, *PCI* percutaneous coronary intervention

A total of 179 consecutive patients undergoing 187 CTO procedures were included in the study (Fig. 1). The clinical characteristics of all patients are summarised in Tab. 1. Mean age was 64.5 years and 74.3% were male. Preserved LVEF was present in 51.9%. In the majority of the patients, non-invasive ischaemia and viability testing was performed. Chest pain was the predominant indication for CTO-PCI (82.9%).

**Table 1:** Baseline characteristics of 179 patients treated with a CTO-PCI

Variables	Number^a^
Age (years)	64.5 ± 11.3
Males	133 (74.3)
Diabetes mellitus	47 (26.3)
Creatine clearance (eGFR)	76.3 ± 19.1
Ischaemia/viability testing performed	133 (74.3)
Left ventricular function (LVEF)	50.6 ± 8.2
Normal left ventricular function (LVEF ≥ 55%)	93 (51.9)
*Medical history*	
– CABG	30 (16.8)
– PCI	99 (55.3)
– Prior myocardial infarction	68 (38.0)
– CVA/TIA	9 (5.0)
Multivessel disease	59 (33.0)

*CABG* Coronary artery bypass grafting, *CTO* chronic total occlusion, *CVA* cerebrovascular accident, *eGFR* estimated glomerular filtration rate, *LVEF *Left ventricular ejection fraction, *PCI* percutaneous coronary intervention, *TIA* transient ischaemic attack

^a^Data are either mean ± SD or number (percentage)

### Angiographic and procedural characteristics

**Fig. 2:**
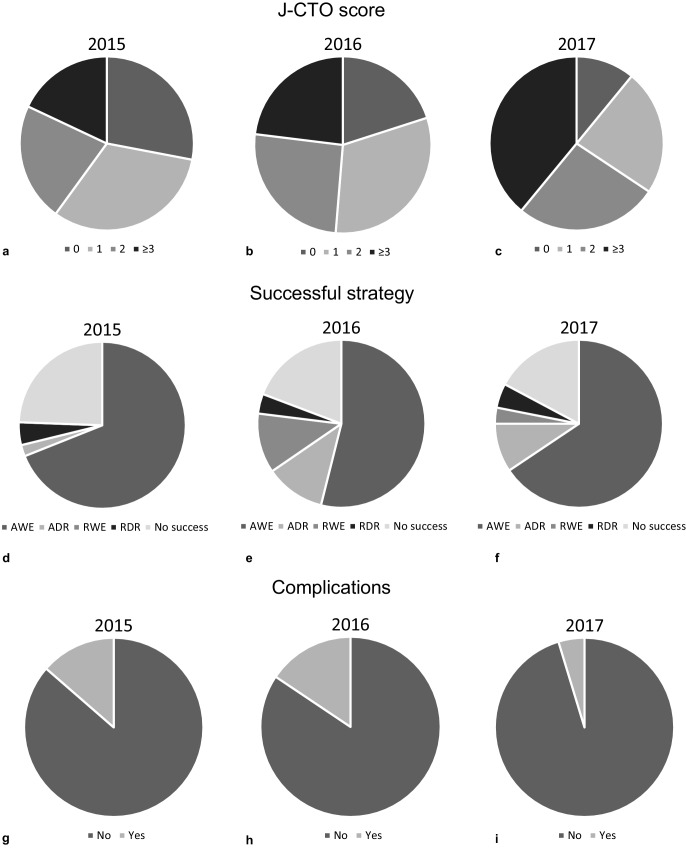
J‑CTO (Multicenter CTO Registry in Japan) score (**a**, **b**, **c**), successful strategy (**d**, **e**, **f**) and complications (**g**, **h**, **i**) during the first 3 years of the chronic total occlusion programme. *ADR* antegrade dissection re-entry, *AWE* antegrade wire escalation, *RDR* retrograde dissection re-entry, *RWE* retrograde wire escalation

Tab. 2 and Fig. 2 summarise the angiographic and procedural characteristics of the complete group for each time period. Over the years the complexity of the CTO lesions increased (from a mean J‑CTO score of 1.3 in 2015 to 2.1 in 2017). The majority of the procedures were performed using the AWE technique. During 2015 a retrograde approach to the distal cap was used in only a limited number of procedures (11.1% as initial strategy, 4.4% as final successful strategy). This increased in 2017 to 17.2% as the initial strategy and 7.8% as the final successful strategy. Final technical success rate was 79.4% in 175 patients with a single CTO (and after repeat procedures in 4 patients), and 80.2% of all 187 CTO procedures (including patients with more than one CTO). The procedural success rates were 77.9% and 79.7%, respectively.

**Table 2:** Angiographic and procedural characteristics of 187 CTO procedures

	Total^a^	2015	2016	2017	*p*-value
*Location*					0.76
LAD	47 (25.1)	8 (17.7)	21 (26.9)	18 (28.2)	
LCx	33 (17.7)	9 (20)	14 (18)	10 (15.7)	
RCA	106 (56.7)	28 (62.3)	43 (55.1)	35 (54.7)	
LM	1 (0.5)	0	0	1 (1.6)	
*CTO lesion characteristics*					
Blunt stump	55 (29.4)	4 (8.9)	24 (30.8)	27 (42.2)	0.001
Calcifications	105 (56.1)	20 (44.4)	47 (60.3)	38 (59.4)	0.26
Bending	64 (34.2)	14 (31.1)	23 (29.5)	27 (42.2)	0.28
Occlusion length	69 (36.9)	15 (33.3)	19 (24.4)	35 (54.7)	0.001
Retry	26 (13.9)	6 (13.3)	13 (16.7)	7 (10.9)	0.67
*Mean J‑CTO score*					
J‑CTO score 0–1	89 (47.6)	27 (60.0)	40 (51.3)	22 (34.3)	0.024
J‑CTO score 2	47 (25.1)	10 (22.2)	20 (25.6)	17 (26.6)	0.90
J‑CTO score ≥3	51 (27.2)	8 (17.8)	18 (23.1)	25 (39.0)	0.024
Mean (SD)	1.7 ± 1.2	1.3 ± 1.1	1.6 ± 1.2	2.1 ± 1.2	0.003
*Successful strategy*					0.46
AWE	115 (61.5)	31 (68.9)	42 (53.8)	42 (65.6)	
ADR^b^	16 (8.6)	1 (2.2)	9 (11.5)	6 (9.4)	
RWE	11 (5.9)	0 (0.0)	9 (11.5)	2 (3.1)	
RDR	8 (4.3)	2 (4.4)	3 (3.8)	3 (4.7)	
No success	37 (19.8)	11 (24.4)	15 (19.2)	11 (17.2)	
*Number of wires used*	5 (3–8)	4 (2–6)	6 (3–9)	5 (3–8)	0.23
*Total stent length (mm)*	48 (33–70)	48 (38–69)	51 (33–67)	48 (26–79)	0.99
*Duration of procedure (min)*	91 (60–139)	75 (54–128)	106 (73–144)	85 (55–120)	0.045
*Total area dose (μGym*^*2*^*)*	9600 (5933–18,500)	10,997 (6661–19,670)	8838 (5939–15,520)	11,218 (5250–19,678)	0.67
*Contrast dose (ml)*	225 (150–350)	180 (120–250)	300 (198–380)	200 (135–280)	0.000

*AWE* antegrade wire escalation; *ADR* antegrade dissection re-entry; *CTO* chronic total occlusion; *LAD* left anterior descending artery; *LCx* left circumflex artery; *LM* left main coronary artery; *J‑CTO* Multicentre CTO Registry in Japan; *RCA* right coronary artery; *RDR* retrograde dissection re-entry; *RWE* retrograde wire escalation

^a^ Number (percentage or interquartile range)

^b^ A total of 5 device-based (2015 = 1, 2016 = 0, 2017 = 4) and 11 wire-based antegrade dissection re-entry procedures were performed

### Procedural complications and clinical outcome

In total, 21 complications occurred during the 187 CTO procedures. There were no peri-procedural or in-hospital deaths (Tab. 3). Coronary perforation occurred in 6 cases, most of which could be managed with prolonged balloon inflations. One perforation was treated with a covered coronary stent and one (more peripheral) perforation with embolisation of fat tissue. This subcutaneous fat tissue was harvested from the patient’s upper thigh and embolised through a microcathether into the distal vessel. Of the patients with a coronary perforation, cardiac tamponade occurred in 2 cases, both managed by pericardiocentesis. No urgent cardiac surgery was necessary. One patient suffered from a peri-procedural MI. In 3 patients a dissection of the donor vessel occurred, which was treated with additional stent placements in 2 patients and in 1 patient conservatively.

**Table 3:** Procedural and in-hospital complications

Variables	*n* (%)	
Total number of peri-procedural complications	21 (11.2)	
*Peri-procedural complications*		
Coronary perforation	6	
– Coronary perforation with tamponade	2	
Septal haematoma	2	
Intracoronary thrombus in donor vessel	2	
Intracoronary thrombus in target vessel	1	
Thrombus in guiding catheter	2	
Dissection of donor vessel	3	
Ventricular tachycardia or fibrillation	3	
*In-hospital complications*		
Death	0	
Myocardial infarction	1	
Stroke	0	
Major bleeding	0	
Urgent cardiac surgery	0	
*30-day follow-up*		
Death	0	
Myocardial infarction	1 (0.6)	
Target vessel revascularisation	1 (0.6)	
*1‑year follow-up*		
Death	4 (2.2)	
Target vessel revascularisation	6 (3.4)	

During the first 30 days no additional MIs and no deaths occurred and only 1 patient underwent TVR (elective coronary artery bypass graft). One-year follow-up data regarding TVR and mortality were available for all patients. After 1 year of follow-up a total of 4 deaths (2.2%) had occurred and 6 patients (3.4%) underwent TVR.

## Discussion

We present the first results of a dedicated CTO programme in a tertiary medical centre. The main finding of this study is that during the first years of the programme the technical and procedural success rates were in accordance with current standards and tended to increase following implementation of the hybrid algorithm. During the same period the complexity of the lesions increased, with only a limited number of serious adverse cardiac events and complications.

To date only three randomised controlled trials have been published on CTO-PCI. The EuroCTO trial randomised 396 patients to OMT versus CTO-PCI and found a significant improvement in health status after CTO-PCI [4]. The EXPLORER trial randomised 304 patients with ST-segment elevation MI and a CTO in a non-culprit vessel to CTO-PCI versus OMT and found no difference in the primary endpoint of LVEF improvement [5]. The recently published IMPACTOR-CTO trial randomised 94 patients with an isolated CTO of the right coronary artery to CTO-PCI versus OMT. Patients with an unsuccessful CTO-PCI and those non-compliant with medical therapy were excluded from the primary analysis. After 12 months of follow-up CTO-PCI was associated with a significant reduction in ischaemic burden as measured by cardiac MRI [6]. The results of the DECISION-CTO trial were recently published, and showed no clinical benefit of CTO-PCI over OMT [7]. However, more methodological issues concerning this study were raised.

Because of the limited number of randomised studies, registries supply additional information on treatment safety and efficacy of CTO-PCI. Since the adoption of the hybrid approach and increased use of retrograde techniques, success rates have increased along with lesion complexity. Several studies have found current success rates between 70% and 85% [2, 12–14]. In accordance with these registry results, we found an excellent rate of in-hospital major adverse cardiac events of less than 1%. Peri-procedural complications were present in around 10%. Although some complications were serious, the majority could be adequately solved during the index procedure without the need for emergency cardiac surgery. One-year follow-up showed excellent clinical outcome in our population. The TVR rate in the studied CTO-PCI population was even lower than the national average for all registered PCIs [15]. With regard to the successful strategies used, AWE remained the predominant strategy during the first years. As AWE remains the basis and start of a CTO-PCI, increased experience and skills have also resulted in more success with AWE techniques.

Regarding the outcome in complex PCIs, it is well known that procedural success and complication rates are strongly dependent on technical skills, operator volume and the availability of dedicated staff and equipment. Current international guidelines emphasise these points, and supply several recommendations regarding training, regional collaboration and volume [7, 8]. Further, they recommend that outcome data are reported by hospitals to national databases to allow outcome monitoring and benchmarking. However, no specific recommendations are given for CTO procedures. The recently published consensus document from the EuroCTO Club supplies more detailed information on centre/operator requirements and expertise in CTO-PCI [16]. They state that centres and operators performing less than 30 CTO procedures annually should refer their patients to a more experienced operator or centre. Retrograde techniques should be reserved for experienced operators performing more than 50 CTO-PCIs per year. A minimum of 50 retrograde procedures might be advised before a cardiologist becomes an independent retrograde operator. These recommendations can be used to draft or extend existing national volume norms for complex PCI.

Our analysis showed that in a high-volume PCI centre it is possible to start a dedicated CTO programme which already fulfils the majority of these volume norms during the first years. Although still a relatively small-scale programme, we expect that with improved success rates, shorter procedure times and increasing numbers of referrals, an increase to a preferred minimum of 100 procedures per year being performed by the dedicated CTO team is most likely in the coming years. In our opinion, the possibility of a joint team from two PCI centres (e.g. one high- and one intermediate-volume) could also be considered.

### Upcoming trials

In the coming years several large randomised clinical trials comparing OMT and CTO-PCI will be performed, e.g. the NOBLE-CTO multicentre trial, which will randomise 2000 patients to CTO-PCI or OMT with an option to cross over after 6 months (NCT03392415). The ISCHEMIC-CTO multicentre trial will randomise 1500 patients to CTO-PCI or OMT (NCT03563417). Also interesting is that a sham-controlled trial is underway: the SHINE CTO single-centre trial (NCT02784418), which will randomise 142 patients to CTO-PCI or a sham procedure.

### Limitations of the study

This single-centre registry study of 179 patients undergoing CTO-PCI suffers from the limitations inherent to this particular study design. The incidence of MIs might be underestimated, as screening by cardiac markers was performed only on indication and not routinely after PCI. Unfortunately, no outcome data were available regarding reduction in ischaemia, improvement in LVEF and symptoms after CTO-PCI.

## Conclusion

Following initiation of a dedicated CTO programme in accordance with current quality and performance guidelines, procedural outcome and complication rates were comparable with the current standards of established centres.

## References

[1] Råmunddal T, Hoebers LP, Henriques JP (2014). Chronic total occlusions in Sweden—a report from the Swedish Coronary Angiography and Angioplasty registry (SCAAR). Plos One.

[2] Christopoulos G, Menon RV, Karmpaliotis D (2014). The efficacy and safety of the “hybrid” approach to coronary chronic total occlusions: insights from a contemporary multicenter US registry and comparison with prior studies. J Invasive Cardiol.

[3] Christakopoulos GE, Christopoulos G, Carlino M (2015). Meta-analysis of clinical outcomes of patients who underwent percutaneous coronary interventions for chronic total occlusions. Am J Cardiol.

[4] Werner GS, Martin-Yuste V, Hildick-Smith D (2018). A randomized multicentre trial to compare revascularization with optimal medical therapy for the treatment of chronic total coronary occlusions. Eur Heart J.

[5] Henriques JP, Hoebers LP, Råmunddal T (2016). Percutaneous intervention for concurrent chronic total occlusions in patients with STEMI: the EXPLORE trial. J Am Coll Cardiol.

[6] Obedinskiy AA, Kretov EI, Boukhris M (2018). The IMPACTOR-CTO trial. JACC Cardiovasc Interv.

[7] Lee SW, Lee PH, Ahn JM (2019). Randomized trial evaluating percutaneous coronary intervention for the treatment of chronic total occlusion. Circulation.

[8] Patel MR, Calhoon JH, Dehmer GJ (2017). ACC/AATS/AHA/ASE/ASNC/SCAI/SCCT/STS 2017 appropriate use criteria for coronary revascularization in patients with stable ischemic heart disease: a report of the American College of Cardiology Appropriate Use Criteria Task Force, American Association for Thoracic Surgery, American Heart Association, American Society of Echocardiography, American Society of Nuclear Cardiology, Society for Cardiovascular Angiography and Interventions, Society of Cardiovascular Computed Tomography, and Society of Thoracic Surgeons. J Am Coll Cardiol.

[9] Neumann FJ, Sousa-Uva M, Ahlsson A (2019). 2018 ESC/EACTS guidelines on myocardial revascularization. Eur Heart J.

[10] Harris PA, Taylor R, Thielke R, Payne J, Gonzalez N, Conde JG (2009). Research electronic data capture (REDCap)—a metadata-driven methodology and workflow process for providing translational research informatics support. J Biomed Inform.

[11] Morino Y, Abe M, Morimoto T (2011). Predicting successful guidewire crossing through chronic total occlusion of native coronary lesions within 30 minutes: the J-CTO (Multicenter CTO Registry in Japan) score as a difficulty grading and time assessment tool. JACC Cardiovasc Interv.

[12] Michael TT, Karmpaliotis D, Brilakis ES (2013). Procedural outcomes of revascularization of chronic total occlusion of native coronary arteries (from a multicenter United States registry). Am J Cardiol.

[13] Habara M, Tsuchikane E, Muramatsu T (2016). Comparison of percutaneous coronary intervention for chronic total occlusion outcome according to operator experience from the Japanese retrograde summit registry. Catheter Cardiovasc Interv.

[14] Konstantinidis NV, Werner GS, Deftereos S (2018). Temporal trends in chronic total occlusion interventions in Europe. Circ Cardiovasc Interv.

[15] NHR (2019). NHR.

[16] Galassi AR, Werner GS, Boukhris M (2019). Percutaneous recanalization of chronic total occlusions: 2019 consensus document from the EuroCTO Club. EuroIntervention.

